# Keep Your Friends Close, but Your Enemies Closer: Role of Acid Sphingomyelinase During Infection and Host Response

**DOI:** 10.3389/fmed.2020.616500

**Published:** 2021-01-21

**Authors:** Ha-Yeun Chung, Ralf A. Claus

**Affiliations:** ^1^Section Translational Neuroimmunology, Department of Neurology, Jena University Hospital, Jena, Germany; ^2^Center for Sepsis Control and Care, Jena University Hospital, Jena, Germany; ^3^Department for Anaesthesiology and Intensive Care, Jena University Hospital, Jena, Germany

**Keywords:** sphingomyelinase (SMase), ceramide (CER), sepsis, organ failure (OF), inhibitor, FIASMA

## Abstract

Breakdown of the inert and constitutive membrane building block sphingomyelin to the highly active lipid mediator ceramide by extracellularly active acid sphingomyelinase is tightly regulated during stress response and opens the gate for invading pathogens, triggering the immune response, development of remote organ failure, and tissue repair following severe infection. How do one enzyme and one mediator manage all of these affairs? Under physiological conditions, the enzyme is located in the lysosomes and takes part in the noiseless metabolism of sphingolipids, but following stress the protein is secreted into circulation. When secreted, acid sphingomyelinase (ASM) is able to hydrolyze sphingomyelin present at the outer leaflet of membranes to ceramide. Its generation troubles the biophysical context of cellular membranes resulting in functional assembly and reorganization of proteins and receptors, also embedded in highly conserved response mechanisms. As a consequence of cellular signaling, not only induction of cell death but also proliferation, differentiation, and fibrogenesis are affected. Here, we discuss the current state of the art on both the impact and function of the enzyme during host response and damage control. Also, the potential role of lysosomotropic agents as functional inhibitors of this upstream alarming cascade is highlighted.

## Introduction—Why Is Consideration of ASM Important In The Context of Infection and Host Response?

During severe infection and sepsis, a stress-responsive enzyme becomes present in circulation, which is known to be essentially involved in membrane repair, internalization of pathogens, maturation of phagolysosomes, and mitochondrial dysfunction. The function of the circulating enzyme is held responsible for rapid and transient formation of the highly bioactive lipid mediator ceramide from the inert membrane constituent sphingomyelin (SM), which is localized at the outer leaflet of cellular membranes. Under physiological conditions, there is a very consequent segregation of the enzyme from its substrate. But why it is important for a better understanding of the pathophysiology of organ dysfunction development, that there is a resolution of the spatial separation? Is there an action of the enzyme for a remote signaling to regulate tissue damage apart from the infectious focus? In this review, we summarize the functions of acid sphingomyelinase in order to contribute to the question whether acid sphingomyelinase (ASM) is our friend or foe in the course of sepsis and severe infection, and further discuss the significance of both, an intended and an unattended inhibition for interpretation of results from preclinical and clinical studies with septic patients.

The first clear description of an enzymatic activity cleaving SM into ceramide with an acidic pH optimum was given by Gatt (1). Twenty-five years later, the protein was isolated and purified from human urine and biochemically characterized (2). Surprisingly, the urine obtained from patients with peritonitis was identified as a rich source for purifying human acid sphingomyelinase (3), which was at that time used as a reagent for *in vitro* experimentation of cellular membranes. Tissue, i.e., brain tissue, was also identified as an appropriate starting material useful for purification procedures (4).

## ASM is Our Buddy

### ASM Profile

The plasma membrane with an asymmetric distribution of phospho- and sphingolipids as well as lateral segregation of SM and cholesterol has—beyond separating cellular compartments—an important function with respect to signal transduction and a plethora of other essential cellular processes (5, 6). The outer leaflet of plasma membranes is enriched in SM, phosphatidylcholines, and glycosphingolipids, whereas in the inner leaflet phosphatidylinositoles, phosphatidylserine, phosphatidylethanolamines, and phosphatidic acid are abundant (5, 7). Metabolism of SM is the entry point in a unique and highly interconnected universe of sphingolipids with a plethora of compounds differing in physicochemical properties and diverse functions regarding cellular signaling and membrane organization (8). The first step—hydrolysis of SM to ceramides and phosphatidylcholine—is catalyzed by the pacemaking enzyme sphingomyelinase, of which so far five different isoforms are known (8, 9). These sphingomyelinases can be distinguished according to primary structure, triggering of activity, cation dependence, and subcellular localization (10, 11). In this review, we focus on acid sphingomyelinase, which is primarily localized in lysosomes (12). Other sources of ceramide formation (*de novo* synthesis, synthesis from sphingosine and fatty acid, as well as hydrolysis of glucosylated or phosphorylated specimen) are playing a minor role during stress response and severe infection (13). A short overview on *in vitro* determination of ASM activity is given in Box 1.

Box 1 **Determination of ASM activity.**

For activity determination, there is a broad range of assays, using either naturally occurring or labeled substrates for *in vitro* measurements up to quenched SM probes: highest structural similarity is given by radiolabelled sphingomyelin (either in the backbone or in acylated fatty acid), when the decrease of SM of formation of ceramide is determined *in vivo* or *in vitro*. This procedure is also suitable for *in-situ* assays without any need for detergent or any other artificial condition [5]. Next, substrates with fluorescently labeled fatty acids in SM were hydrolyzed by ASM, the generated corresponding ceramide derivative is separated using thin-layer chromatography TLC and determined using a CCD camera in a high-throughput format [6]. In addition, SM substrates with radioactively labeled phosphocholine are used following extraction of the water-soluble reaction product [7]. An artificial SM substrate with short chain fatty acids are used with highly specific mass spectrometric analysis of corresponding ceramides resulting in improved sensitivity [8]. From naturally occurring sphingomyelin, another opportunity for determination of the second reaction product (phosphocholine) is oxidation of a pro-fluorescence dye following hydrolysis and oxidation to betaine, where formation of a resorufin analog by released hydrogen peroxide is used as a reporter system. The strength of this assay format is the transfer to a multi-well-format without need of any separation step [9]. A most recent review on strengths and pitfalls of ASM assays is given by Nikolova-Karakashian [10].

Quenched fluorescent SM probes based on FAM/BODIPY dyes are allowing real-time analysis to monitor relative sphingomyelinase activities and ceramide formation of intact, living cells by techniques of flow cytometry. At the end, these probes are acting as a biosensor in a non-invasive manner and in native cellular environment with high spatial and temporal resolution [11, 12]. The specific profile of generated ceramides in affected cells is quantified by methods of mass spectrometry following lipid extraction and chromatographical separation [13].

Almost 30 years ago (1991), the full sequence of ASM was firstly described (14, 15). The genetic locus was identified on the short arm of chromosome 11 (15), interestingly in close proximity to the locus of other lysosomal proteins such as cathepsin D and acid phosphatase (13). The sequence of ASM is highly conserved among mammals (16) and the ASM locus [systematically sphingomyelin phosphodiesterase 1 (SMPD1)] is undergoing epigenetic regulation by paternal imprinting (17). During protein synthesis, ASM undergoes extensive posttranslational modification. In this process, glycosylation at all of the six potential sites is of major importance to ensure correct folding, sorting, and/or proper stability within the proteolytic milieu of the lysosome (18, 19). By mannose-6-phosphate-receptor shuttling, the protein is transported via the endosomal system to its final destination, the lysosome (20).

Along the primary subcellular localization, the purified protein favors an optimum pH around 5.0, but sphingolytic activity is retained also at neutral pH (21): regarding parameter of enzymatic activity, despite the pH shift up to 7.45, the maximum turnover velocity (*v*_max_) remains constant (22, 23). The decrease in affinity to the substrate (*K*_m_) is of no relevance due to the enormous excess of SM at the outer leaflet of cell membranes (24, 25). The persistence of enzymatic activity at neutral pH values is of great importance as discussed later.

*In vitro* experiments showed us that enzyme activity is also dependent on lipid environment (26) and addition of a detergent in order to overcome dependence from activation by specific proteins (26). Sensitivity to reducing agents such as dithiothreitol is underlining the relevance of disulfide bridges within the mature protein structure for hydrolyzing activity (27, 28).

Resolving the crystal structure of mammalian ASM (with 88% identity to the human protein) confirmed that ASM-mediated hydrolysis of SM is functioning in a canonical mechanism, where phosphoesterases are utilizing a nucleophilic attack of a zinc (Zn^++^)-activated water molecule and protonation of the leaving group for release of phosphocholine and ceramide (29). Two saposin domains are relevant for determination and stabilization of either a closed or open conformation of the enzyme. In the latter one, the enzyme is able to bind and dock to membranes, and extract SM therefrom for subsequent hydrolysis (29). Direct inhibitors of the enzyme (i.e., bisphosphonic acid derivatives) are competing with Zn^++^ binding within the active center (29). A short overview on milestones in translational research regarding ASM activity is given in Table 1.

**Table 1:** Milestones for ASM in translational sepsis research.

**Finding**	**Year**	**References**
First observation of sphingomyelin hydrolysis at acid conditions	1963	(1)
ASM deficiency as molecular basis of Niemann-Pick disease, types A and B (NPD-A and NPD-B, respectively)	1966	(30)
Purification of ASM from urine (obtained from septic patients)	1987, 1989	(2, 3)
Description of a Zn^++^-dependent secreted isoform of ASM	1989, 1996	(31, 32)
Sequencing, cloning, and locus mapping of full-length ASM	1991	(14, 15)
Generation of ASM-deficient mice as NPD model	1995	(33)
Association with severity in septic patients	2005	(34)
Phosphorylation of S508 for activation and translocation	2007	(35)
Cationic amphiphilic drugs as functional inhibitors of ASM (FIASMA)	2010	(36)
Crystal structure of mammalian ASM	2016	(29)
Triple combination of FIASMA blocking Ebola virus infection	2017	(37)
Re-evaluation of the puzzle	2019	(38)

*Description of milestones, which are mostly relevant for a better understanding of the role of ASM during host response, development of organ failure, and proposed treatment thereof*.

Under physiological conditions, ASM is fulfilling essential house-keeping functions in the lysosomes, whereas genetic deficiency leads to extensive accumulation and deposition of SM resulting in organ abnormalities as described in Niemann-Pick disease (NBD), types A and B (30). The moment of glory has come to ASM as soon as response to external or internal signals of stress is much needed. Since ceramide generation is the common final pathway of most stressors, the panel of ASM stimulators in a large variety of cell types goes far beyond those previously described for any other molecular switch of the SM pathway and are summarized in Table 2. Activation of the ASM-sensitive pathway leads to a rapid and transient translocation of the enzyme toward the cell membrane ranging from seconds to hours (60, 61).

**Table 2:** Stressors triggering ASM activity and translocation (selection).

**Stressors and agonists**		**References**
Pathogens	Viral (rhinovirus, Ebola, SinbisV)	(39–42)
	Bacterial (*Neisseria gonorrhea, Staphylococcus aureus, Pseudomonas aeruginosa*)	(43–49)
	Parasitic (*Cryptosporidum parvum*)	(50)
Endogenous danger signals	Cytokines (IL1-β, TNF-α)	(51, 52)
	Ligation of death receptors (TNF-α, CD95, TRAIL)	(13)
Cytotoxic agents/drugs	PMA, *cis*-platin, paclitaxel, retinoic acid, doxorubicin	(35, 37, 53–55)
(Chemotherapeutics)	Rituximab	(56)
Radiation	UV-C, ionizing radiation	(57)
Oxidative stress	Ischemia/reperfusion injury	(58)
	Generation of reactive oxygen species	(44, 45, 59)

*Outline of stressors and harmful events resulting in triggering extracellular ASM activity by translocation and exocytosis of the lysosome with decompartimentalization and amplification of biological effects of ASM at the outer leaflet of cellular membranes (selection)*.

In order to highlight the importance of ceramide generation in the course of a severe systemic disease, it is noteworthy that ASM-deficient fibroblasts or mice are resistant to radiation-induced cell death (57). In addition, specific ceramide-binding antibodies rescued mice from lethal radiation gastrointestinal syndrome by preventing signaling platform formation (see below) and inhibition of endothelial cell death (62).

### Maturation of Phagolysosome, Translocation, and Role in Raft Modeling

ASM is essential for proper fusion of late phagosomes with lysosomes, which is crucial for efficient transfer of lysosomal antibacterial hydrolases into phagosomes (63, 64). A significant role of ASM in the phagolysosomal compartment for the defense against infection with intracellular pathogens was shown by a dramatically increased susceptibility to *Listeria monocytogenes* in ASM-deficient mice (ASMKO). Although ASM-deficient immune cells showed intact production of reactive nitrogen intermediates and oxidative burst, they are completely incapable of restricting and controlling the intracellular growth of *L. monocytogenes in vitro* (65). Similar findings were obtained from mouse peritoneal macrophages infected with the obligate intracellular protozoan, *Leishmania donovani*. Increase of intracellular ceramide was not only a consequence of ASM-triggered activity but also from *de novo* synthesis, which resulted in upregulation of Ca^++^-independent atypical protein kinase C (PKC)-ζ. Surprisingly, suppression of formation of reactive nitrogen species (i.e., nitric oxide) facilitated the survival of leishmanial parasites in the intramacrophageal milieu (66).

Accumulation of reactive oxygen species (ROS), as a consequence of altered redox status followed by ASM activation, ceramide generation, and subsequent clustering of CD95 in ceramide-enriched lipid rafts is a common and early event in neutrophil apoptosis, which are abundant, and short-lived leukocytes. Their death by apoptosis is central to hemostasis and the resolution of inflammation (67).

### Extralysosomal Activity—Circulation in Plasma?

In 1996, Tabas et al. described an additional, plasma-secreted, circulating product of the sphingomyelin phosphodiesterase 1 (*SMPD1*) gene with a similar glycosylation pattern, but increased dependency and susceptibility to Zn^++^ ions (31, 68). Differential protein trafficking was held responsible for the regularly observed activity increase in men, mice, and cell culture experiments upon stimulation, but the underlying mechanisms remain unknown. There is an ongoing debate on the origin of the ASM isoform circulating in plasma, since the mechanisms for a differentiated intracellular trafficking upon stimulation are hard to explain. Phosphorylation of serine residue 508 (S508) by PKC-δ upon stimulation with phorbol ester or UV light was considered to be essential for activation and translocation (35). But to the best of our knowledge, there is no evidence of phosphorylated ASM in disease models or clinical samples.

**Figure 1:**
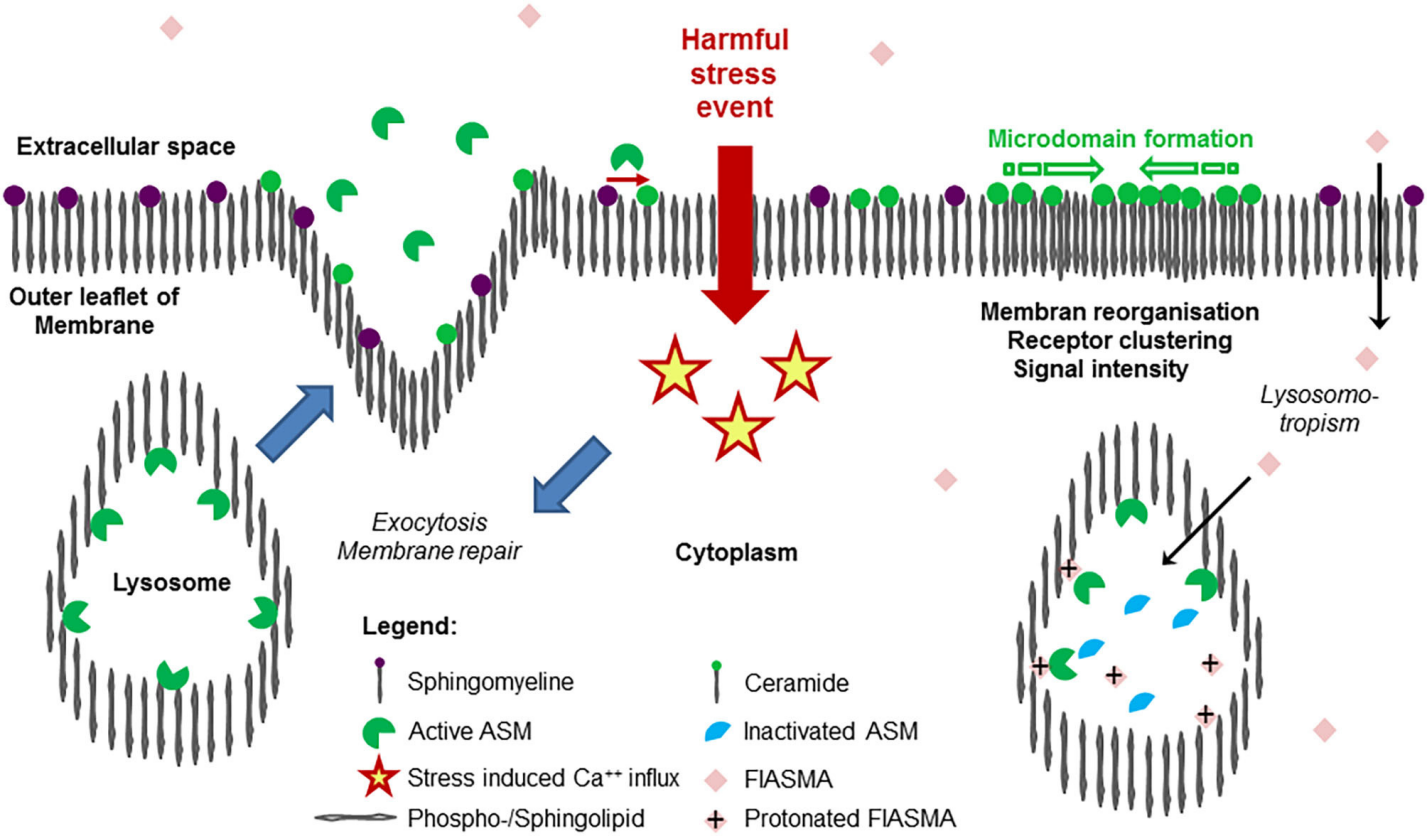
Release of acid sphingomyelinase (ASM) in the course of stress response and mode of action of its inhibition. Exposition of a cell membrane to external harmful stress events such as endogenous or exogenous stimuli (bacterial endotoxin, pro-inflammatory cytokines, pore-forming toxins, Table 2) is followed by intracellular Ca^++^ influx, triggering the exocytosis of lysosomes, where ASM was bound to the inner lysosomal membrane by SAP domain. In the extracellular space, ASM mediates hydrolysis of SM (abundantly embedded into the outer leaflet of the membrane), generates ceramide, facilitating remodeling, and repair of the membrane (restoring integrity), as well as pathogen entry. As a hallmark of ceramide-induced signal transduction, due to the trend for self-aggregation and formation of ceramide-enriched microdomains, subunits of receptor proteins are reorganized to functionally active receptor complexes such as TLR4, TNFR, etc. As a result, an increase in signaling quality and intensity is observed, controlling an adequate cellular response to external harmful stimuli. On the right panel, the mode of action of cationic amphiphilic substances is illustrated: in an uncharged form at physiological pH value, the compounds diffuse across the cellular membrane through the cytoplasma into the lysosome, where the weak basic nitrogen atom of the compound is protonated because of the acid pH value of the lysosome. The protonated compound interacts with the sapsonin domain of ASM, detaching them from the membrane, then undergoing proteolytic inactivation. Following stimulation and release, the inactivated ASM is unable to contribute to ceramide generation at the outer leaflet of the membrane. Due to similar physicochemical properties, a long list of cationic amphiphilic substances with a broad range of clinical indications, but most of them used in daily care as antidepressive drugs, are found to function as functional inhibitors of acid sphingomyelinase (FIASMA) and to effectively control stress-induced ceramide generation. Most of the available FIASMAs are licensed for medical use in humans, are minimally toxic, and may therefore be applied for disease states associated with increased activity of ASM.

However, recent studies with the protozoan parasite *Trypanosoma cruzi* revealed that conventional lysosomes are regularly fusing with the plasma membrane in response to increased intracellular Ca^++^ concentration with subsequent triggering of exocytosis (69) and release of the intralysosomal content to the extracellular space, eventually also into circulation. As a result, exocytosed ASM is capable to act at the outer leaflet of the membrane. This mechanism is thought to define the role of extracellular ASM as well as to represent the major source of extracellular form of the protein (38). The findings, that a plethora of harmful, stress/injury-triggering events induced lysosomal exocytosis including interaction with pathogens (59, 70, 71) offers a plausible perspective of the possible origin of extracellular and circulating ASM activity without any need for postulating differential trafficking (38). An overview on ASM release as a feature of stress response is given in Figure 1.

**Figure 2:**
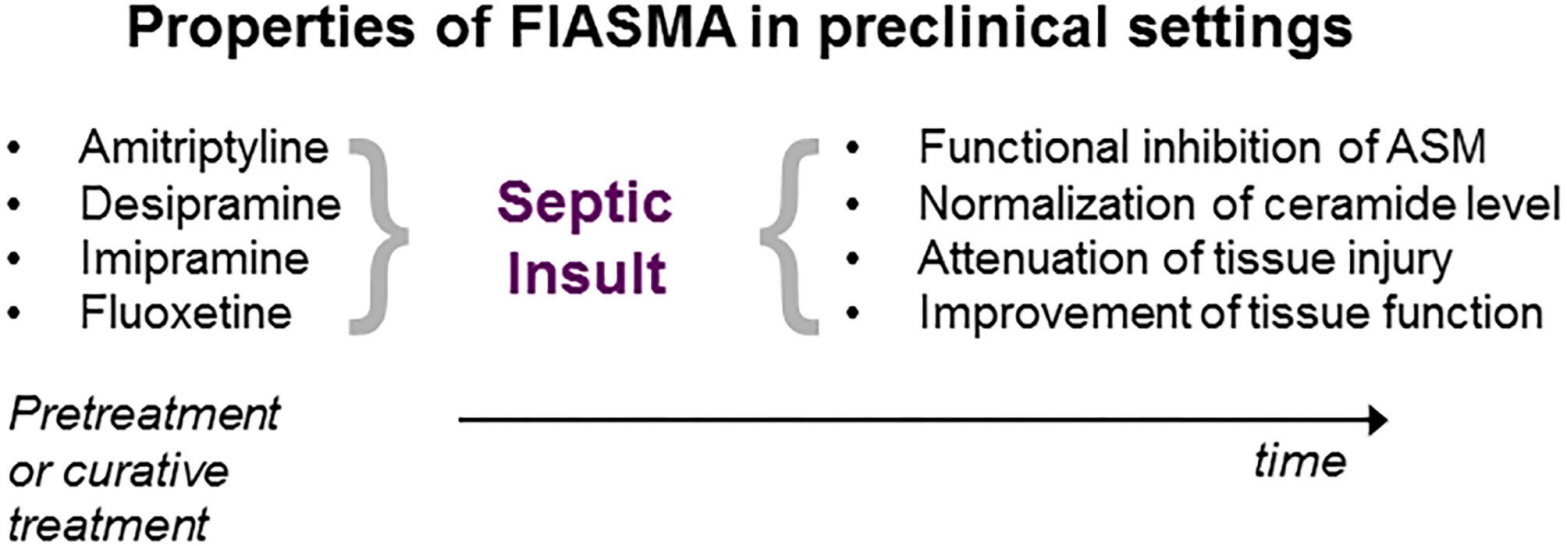
Properties of ASM inhibition in preclinical sepsis research. Administration of the inhibitor either prior (pretreatment) or following septic insult (curative). For details, see text.

Exposition of immune cells to ROS resulted also in a rapid and transient, Ca^++^-dependent translocation of ASM to the outer leaflet of cellular membranes—mediated by exocytosis of lysosomes—and formation of ceramide-enriched platforms (59). On the other hand, these microdomains are required for *Pseudomonas aeruginosa*-induced activation of NADPH oxidase and production of ROS, demonstrating a positive feedback mechanism for amplification of ASM-mediated redox signaling (43).

On a molecular level, the generation of ceramide is an integral part of intrinsic repair mechanisms following perforation of the plasmalemma by pathogenic pore-forming toxins, whereby lysosomes fuse with the plasma membrane. Hereby, lysosomes externalize their contents including acid sphingomyelinase and facilitate exposure to the abundant SM at the outer membrane bilayer (70, 72). Subsequent formation of ceramide-enriched, topically restricted domains in the outer leaflet of the lipid bilayer is an essential step contributing to tighter packing of the membrane, forwarding a negative curvature and inward vesiculation of the damaged area. At the end, the resulting internal degradation contributes to cellular integrity and survival (72, 73), e.g., after exposition of cells toward listeriolysin O or pneumolysin (74).

### Microdomain Formation, Protein Organization, and Ceramide Signaling

In cultured immune cells, exposure to endotoxin led to activation of ASM, generation of ceramide, phosphorylation of PKC-ζ, assembly of Toll-like receptor 4 (TLR4) within lipid rafts, activation of the stress-responsive kinases, and release of tumor necrosis factor-α (TNF-α). These pathogenetic mechanisms could be abrogated by CD14 blockade or inhibition of ASM but reversed by treatment with the central effector molecule ceramide (51). In addition to other proteins associated with the lipid raft, ASM is contributing to TLR4 signaling triggered by endotoxin and non-microbial endogenous ligands (75) (Figure 1).

Moreover, ASM is involved in most effective host membrane remodeling during enteropathogenic infection with *Shigella* spp. resulting in decreased binding of pathogens to epithelial cells and therefore, impeding abovementioned or other pathogens from reinfection, which was proposed as a novel stress-responsive cell-autonomous defense mechanism (76).

In concert with the endogenous danger signal adenosine triphosphate (ATP), ASM is involved in secretion of membrane TNF-α within microvesicles bypassing conventional pathways. These cytokine-carrying microvesicles are biologically more potent than soluble TNF-α *in vivo*, evolving significant lung inflammation in mice, which might have crucial implications for the biological activity of this prototypically proinflammatory cytokine (77).

### Ceramide as Second Messenger

Beyond the membrane reorganizing capacities (78), ceramides also act as second messengers transducing cellular signals (79). These functions widely differ among the diverse cell types, ranging from induction of senescence to apoptosis. As an example, the role of ceramide generation and the function of generated ceramides in mitochondria were extensively studied (80): organelle-specific accumulation of ceramide is critically involved in progression of mitochondrial apoptosis and mitophagy, defining ceramides as a *bona fide* transducer of mitochondrial (dys-)function [excellently reviewed in (80) and references cited therein].

In the next paragraphs, consequences of ASM depletion in the course of local or systemic infections with the presentation of interesting phenotypes will be discussed. In 1995, the generation of ASM-deficient mice was of great value for studying the pathogenesis and treatment not only of type A/B-Niemann-Pick disease (see below) but also for investigations into the role of ASM in ceramide signaling, induction of apoptosis, and ceramide-induced tissue damage (33).

### Hyperresponsiveness

Mice, genetically lacking ASM (33), showed an exaggerated response to polymicrobial sepsis with an increased bacterial burden, an enhanced phagocytotic activity, a more pronounced cytokine storm and decreased survival rate (81). Moreover, on a functional level, leukocyte-endothelial interaction was found diminished in ASMKO animals corresponding to a distinct leukocytes' phenotype with respect to rolling and sticking as well as expression of cellular surface proteins (81). A similar phenotype was found in cultivated lung epithelial cells with controlled ASM activity, where increased neutrophil recruitment, elevated levels of cytokine mRNA, and a pro-oxidative shift could be observed (82).

Moreover, the availability of ASMKO mice provides the opportunity of a better understanding of ceramide generation for immune response on a molecular level. In a fundamental experimental setting, injection of endotoxin and its putative effector TNF-α, into mice induced disseminated apoptosis in endothelium of intestine, lung, fat tissue, and thymus accompanied with cytokine release and ceramide increase. This endothelial cell death was ASM dependent, since ASMKO mice were protected against endothelial apoptosis and animal death (83).

In mice and men, complete loss of function of ASM resulted in a diminished CD1d-restricted antigen presentation of invariant natural killer cells (iNKT), decreased levels of this cell population and resistance to iNKT cell-mediated inflammatory conditions, supporting the concept of a tight link between cellular sphingolipid metabolism and immunity (84).

### Pneumonia in ASM-Deficient Patients

Niemann-Pick disease (types A and B) is an autosomal recessive lysosomal storage disorder caused by biallelic mutations in the *SMPD1* gene resulting in a severe human disease state characterized by deficient ASM activity (85). A recent study reported around 185 mutations with ASM-deficient NPD worldwide (86), while the detection of new mutations is not yet completed (87). The majority of mutations are predicted to affect proper folding, stability, as well as membrane binding of the enzyme (29). Type B patients are characterized by hepatosplenomegaly and progressive alterations of the respiratory system, but the central nervous system is usually less affected, which is more profound in type A, resulting in early death (~2 years of age) (85). Lung involvement is the most important prognostic factor in NPD-B, with recurrent respiratory infections starting in infancy being the major cause of morbidity and mortality (82, 88, 89). In lung epithelial cells, decreasing ASM activity by 50% leads to an increased neutrophil recruitment via elevated levels of cytokine expression, both at baseline and in response to bacterial stimulation. Instead of preventing the host defense responses, decreased ASM activity results in an inflammatory response even in the absence of infection supporting the hypothesis of a chronic inflammatory state impairing host defense mechanisms (82). Despite the low incidence of progressive pulmonary disease in a cohort of more than 100 patients diagnosed with NPD-B, pneumonia was the leading cause of death of juvenile patients (<21 years) (90, 91). On the one hand, pathophysiology of the pulmonary disease is presumably related to the accumulation of SM in alveolar macrophages, on the other hand, inflammation, abnormal surfactant catabolism, as well as composition contributing to lung abnormalities was shown in ASMKO mice (92, 93). In parallel, endogenous lipid pneumonia, interstitial fibrosis, and accumulation of foamy macrophages were found in human lung biopsies (94) supporting the hypothesis that the lung is a primarily affected organ of NPD-B contributing to morbidity and mortality (90, 95).

In a brief summary of this section, ASM is a highly conserved stress-responsive enzyme, activity of which is triggered by a variety of harmful events including infection and inflammation. As a key consequence of activation, there is a disorganization of the previously strict segregation of the enzyme (located in the lysosome) and the corresponding substrate (SM in the outer leaflet of cellular membranes). Besides switching the current pH status by two orders of magnitudes, the enzyme is capable of rapid and transient ceramide formation upon activation. The resulting self-aggregation of ceramide molecules is reorganizing cellular membranes. Now it is time to put this critical event of signal amplification as a general mechanism into the scene of stress response, membrane repair, oxidative stress, proper maturation of phagolysosomes, and regulation of host defense against invading pathogens. As our buddy—keeping us well and fine by its integration in imperative actions of life—the enzyme and its regulation of translocation and activity received also value for further elucidation with respect to pathophysiology and pathogenesis of a series of diseases.

## ASM is A Dubious Friend

### Invasion of Pathogens

Non-human sphingomyelinases are essential factors for virulence of extracellular, facultative, or obligate intracellular pathogens. These enzymes contribute to phagosomal escape or phagosomal maturation avoidance and even immune response evasion (96). Whether the activity profile of these prokaryotic proteins contribute to the analyzed hydrolysis rate of SM to ceramide in conventional assays in samples obtained from patients with blood stream infection (Box 1) is still discussed controversially. Some prominent and representative mechanistic approaches are shown here in an exemplary manner.

Infections by *Staphylococcus aureus* are a major clinical problem ranging from mild infections (skin and soft-tissue) to severe and even lethal infections (e.g., pneumonia, endocarditis, sepsis, osteomyelitis, etc.). *S. aureus* stimulates ASM via CD44-triggered release of ROS, resulting in ceramide release, clustering of CD44 in ceramide-enriched membrane platforms, co-activation of GTPases, and translocation of linker proteins with subsequent rapid rearrangement of the cytoskeleton. In the absence of either CD44 or ASM, reduced internalization of macrophages is counteracted by a reduced killing capacity (44).

A major toxin of *S. aureus* (α-toxin) caused in bone marrow-derived macrophages ASM-dependent ceramide formation, release of cathepsin B and D from lysosomes, inflammasome activation, and induction of pro-inflammatory cytokines, which could be abrogated by pharmacological ASM-inhibition (45). In *in vitro* experiments with endothelial cells, ASM activation by α-toxin was linked to degradation of tight junctions, which could be blocked by pharmacological inhibition. However, most importantly, in *in vivo* experiments, genetic deficiency prevented severe degradation of tight junctions in the lung and edema formation (46). Combination of antibiotic treatment and ASM inhibition are proposed to exhibit synergistic and super-additive effects (47).

Inflammasomes are important for host defense against invading pathogens; ASM activation is critically involved in the activation of endothelial inflammasomes including recruitment of adapter proteins and caspases (97–99) and subsequent oxidative signaling by lipid raft-associated redox platforms, release of cytokines, activation of stress kinases, and altering tight junctions in epithelial cells (45).

For exposition of proteins, non-enveloped viruses undergo partial uncoating in order to get access to the cytoplasm by membrane lysis. Cellular ASM is a critical player in this process, since ASM is induced and hijacked by mimicking wound removal processes facilitating the adenovirus further membrane disruption and infection. In addition, stimulation of Ca^++^ influx and lysosomal exocytosis are key steps for efficient membrane penetration of the virus (71). Most recently, influenza A virus infection was found to be associated with suppression of ASM activity in cultured alveolar epithelial cells, whereby depletion of SM content either in the epithelial membrane or in the virus envelope impaired virus infection and reduced virus entry as well as reduced virus infectivity and impaired its attachment and internalization, respectively. Inhibition of ASM by desipramine did not affect influenza virus infection (100), which is in line with the observation that ceramide generated by *de novo* synthesis might play an antiviral role (101).

For meningococcal internalization into brain endothelial cells, transient ASM activation and ceramide release are also determinative factors for invasiveness among a defined set of pathogenic isolates of *Neisseria meningitidis* (102).

In the pathogenesis of cystic fibrosis, abrogation of ceramide accumulation by inhaled functional inhibitor of ASM (FIASMA, see below) restored normal ceramide concentrations in murine bronchial epithelial cells, reduced age-dependent pulmonary inflammation and deposits of DNA in bronchi, as well as prevented infection with *P. aeruginosa* (103, 104). ASM activation (by endotoxin) and subsequent ceramide formation play a pivotal role in *Escherichia coli*-induced apoptosis of immature dendritic cells, a phenomenon contributing to sepsis-induced immunosuppression (105). In liver specimen, inhibition of activity in endotoxin challenged mice correlated with a reduced rate of hepatocellular apoptosis (34).

It is also known that ceramide-containing microparticles in packed red blood cells contribute to adverse effects following transfusion, which was abrogated by treatment with a FIASMA during storage time (106).

ASM activation is a critical factor for redirection of TNF-α trafficking, thus the cytokine is embedded in microvesicles bypassing conventional pathways in a highly potent manner. Rerouting has crucial implications for the activity profile of the pacemaker cytokine of inflammation, inducing a significant TNF-dependent inflammation status and allowing long-range TNF signaling to target cells more effectively than soluble TNF, which is of particular interest therapeutically targeting TNF in acute inflammatory diseases (77).

### Marker and Mediator in Sepsis and Pneumonia

Endotoxin challenge in mice resulted in a mild, 2-fold increase of ASM plasma activity, which was accompanied by release of cytokines (107). These data were confirmed by a similar observation of an increase of ceramide content in lipoproteins paralleled by an increased activity of circulating ASM (108).

Intratracheal administration of an ASM inhibitor in a model of acute lung injury improved lung function and decreased pulmonary inflammation (109). In critically ill preterm infants with acute lung injury, beyond other markers and mediators, ASM activity was found to be increased in tracheal aspirates (110). Inhibition of NF-κB function attenuated pulmonary inflammation of acute respiratory distress syndrome in a neonatal piglet model with decreased leukocyte concentrations in bronchoalveolar lavage, reduced ASM activity, and subsequently decreased ceramide levels (111). This emphasizes the pivotal role of sphingolipid signaling controlling pulmonary edema formation and lung function.

In patients with community-acquired pneumonia (CAP), a plasma decrease of SM was found to be closely associated with an increase in ceramides (16:0, 18:0, 24:1), but normalized toward clinical remission (112). Furthermore, the sphingolytic activity of plasma-secreted ASM was nearly 3-fold increased with same tendency for normalization. A similar course was also observed with respect to gene expression rate of SMPD1 in circulating leukocytes, supporting the concept of a disease-relevant regulation of ASM expression in CAP at both, protein and mRNA level. These observations might qualify ASM as a potential target for host-directed treatments to reduce end-organ damage in pneumonia (112).

### Endothelial Integrity

Cultivated endothelial cells are affected by serum obtained from patients with sepsis, resulting in clustering of receptors relevant for signal transduction and suppression of a stress-sensitive transcript marker of these cells (i.e., ADAMTS13), which is abrogated by direct and indirect inhibition of ASM activity (113). Both, plasma activity and amount of ASM were found to be increased in septic patients dependent on clinical severity (34, 113). Thus, ASM is involved in the dysregulation of ceramide metabolism in endothelial cells leading to macrodomain formation, cytotoxicity, and downregulation of ADAMTS13 expression (113), which is held to function as an adverse effect to endothelial dysfunction and microthrombus formation in sepsis (114). These results obtained in *in vitro* experimentation were recently confirmed in a monocentric clinical study, where a plasma decrease of the endothelium-stabilizing mediator sphingosine-1-phosphate (S1P) was strongly negatively associated with an increase in ceramide levels (115). Moreover, the association with severity of clinical course could be outlined by a highly powerful value of an integrative analysis of the S1P/Cer ratio for prediction of unfavorable outcome, superior to established severity scores (SOFA) and HLA-DR expression on circulating monocytes (115).

### Role of Alternative Splicing and SNP in Sepsis and Depression

Beside predominant localization in lysosomes especially of monocytic, hepatic, and endothelial cells, and delocalization of the protein to the outer leaflet of cellular membranes in response to multiple stimuli including pathogens (116), encoding mRNA_SMPD1_ is undergoing alternative splicing as an independent matter of stress response. A series of *SMPD1* mRNA splice isoforms are described to date, but only ASM-1 as the full-length transcript, Ref_mRNA NM_000543 has been shown to be translated into a catalytically active protein (14, 117, 118). It might be speculated, that the extensive variability is a marker of negative regulation with highest variability in healthy controls but decreasing in a stress condition such as severe infection by skipping of exon 3 and/or elongation of exon 2 (119). Thus, an association of specific pattern of alternatively spliced *SMPD1* transcripts with disease severity (healthy and non-infectious patients undergoing intensive care as controls vs. sepsis) was observed (119). At the end, alternative splicing of *SMPD1* might act in a dominant negative manner upon overexpression making alternative splicing a promising target in regulation of ASM activity (118, 119). Interestingly, a similar phenomenon was observed in patients with major depression, a disease, where despite clear associations of ASM activity with the severity of the disease (120) the regulatory mechanisms and cause-effect links are not well-understood (121). Strikingly, alternative splicing of SMPD1 was also found to be reduced in depressed patients, and most interestingly, normalized upon subchronical treatment with FIASMA in both, patients and healthy volunteers (118, 122).

There are a small number of reports on polymorphisms of the *SMPD1* gene locus: V36A, A487V, and G508R as well as hexanucleotide repeat in the signaling peptide (123–127). In contrast to the variety of missense mutations, there is only a minor effect of these sequence variations on overall activity and function of the enzyme, but they might increase the susceptibility for common diseases such as allergy (125). The G508A transition—exchange of an uncharged with a charged amino acid—is discussed to be significant for recognition of a potential phosphorylation site, which is relevant for the control of protein activation and secretion (35, 52). In line with this hypothesis, G508A is associated with plasma-secreted activity in a gene-dosage-dependent manner (125), since subjects homozygous for the minor A allele displayed half of the plasma activity compared with the major G allele. Whether this observation on a molecular level might contribute to control the susceptibility to infections is still a matter of debate, but as discussed below, a decreased ASM activity is associated with lower respiratory tract infections (90, 91).

In brief, ASM is also held responsible for internalization of pathogens (bacteria and viruses). In acute and chronic infection, inhibition of ASM improved outcome in (pre-)clinical studies in patients with cystic fibrosis and CAP. ASM inhibition also diminished inflammation-responsive fibrogenesis following sepsis in a preclinical study. A multilevel regulation with association to favorable outcome was observed in patients undergoing CAP. Due to the facts that (i) the impact of single nucleotide polymorphisms and alternative splicing of ASM with respect to susceptibility and progression of infection is not fully understood and (ii) pharmacological inhibition of the enzyme resulted in improved outcome from chronic infection, the role of ASM should not be evaluated as exclusively favorable without any restriction. Our friend is becoming dubious and needs more critical consideration.

## But ASM is Also Worrying

In a series of clinical and preclinical studies, ASM activity was positively associated with the severity of the disease. In a small, mixed population from an intensive care unit (ICU), ASM activity was found to be increased and to remain elevated in the presence of a low level of procalcitonin-discriminating non-surviving patients after systemic inflammation. Also, a posttrauma effect with a significant increase in ASM after surgery parallel to a postoperative increase of procalcitonin and C-reactive protein was observed (128). Strikingly, patients with severe sepsis exhibit an enhanced, 2-fold sphingolytic activity in comparison with controls. A further increase was associated with greater risk by the severity of illness and with fatal outcome (34). Supporting the hypothesis that ASM activity is highly correlated to inflammation, we also found ASM activity mildly increased in patients with rheumatoid arthritis, which was associated with oxidant activity, markers of inflammation, and endothelial activation (129). Interestingly, treatment of these patients by biologicals with TNF-α-binding capacity suppressed the increase completely to levels as found in healthy controls along with improvement of the clinical condition (130). These data support the concept that activation of circulating ASM may play a critical role in the development of apoptosis and organ failure in inflammation-associated disease, especially sepsis. An inhibition of ASM should be explored further as a potential target in the complicated puzzle of sepsis. At sepsis diagnosis, leukocyte-associated ceramide content as a result of ASM activity was significantly elevated and correlated to TNF-α concentration as both a marker of cytokine release as well as mediator of ASM activation (131). Ceramide concentration was highly predictive for risk of development of organ failure (131).

In hemophagocytic lymphohistiocytosis (HLH), a rare systemic inflammatory syndrome resulting from unrestrained immune cell activation, levels of circulating ASM were found elevated. Also, there was a shift in ceramide and sphingosine ratio (increase), while levels of S1P were decreased (132). Interestingly, an elevated ratio between ceramide and sphingosine was predictive for unfavorable outcome (132). Data were confirmed in a small cohort with a 10–20-fold increase of ASM activity with a trend to normalization during recovery (133).

As an indirect measure for ASM-induced unfavorable outcome, fluoxetine and desipramine reduced in an lipopolysaccharide (LPS)-induced model of septic shock the levels of circulating TNF-α similar to prednisolone, accompanied with an improved outcome to untreated controls (134). In murine models of peritonitis and endotoxemia following LPS challenge, amitriptyline-treated mice were protected from overwhelming cytokine release (KC, MCP-1) and from pulmonary edema as well as exhibiting improved survival. Anti-inflammatory effect of amitriptyline treatment is reflected by increased IL-10 levels and decreased accumulation of immune cells at the site of infection (135).

### Eryptosis

In patients with hyperbilirubinemia, as often observed in the acute phase of severe infection due to sepsis-induced cholestasis and also a long-term consequence of survivors (136, 137), high levels of conjugated bilirubin may also lead to progressive sclerosing cholangitis (138) and are able to stimulate suicidal death of human erythrocytes. As an underlying mechanism, bilirubin triggered rapid Ca^++^ influx resulting in the release of ASM, formation of ceramide, and subsequent translocation of phosphatidylserine to the erythrocyte surface (139), a phenomenon, which is also compatible with the anemia status of these patients exerting an increased mortality rate (140).

### Platelets

Ceramide accumulation over time, generated by ASM in stored and aged platelets, caused lung injury in endotoxin-challenged mice, examined by neutrophil accumulation, endothelial barrier dysfunction, and histological evidence of tissue injury (141). Interestingly, this adverse effect of pulmonary complications following transfusion could be overcome by ASM inhibition (141).

### Mechanisms and Effects of ASM Inhibition

A broad panel of cationic amphiphilic compounds is known to inhibit the activity of ASM by lysosomotropism due to their unique physicochemical properties, which was firstly observed by Albouz et al. (142). Interaction with the membrane-embedding *N-*terminal saposin domain of ASM caused detachment from the inner lysosomal membrane and a consecutive proteolysis of the enzyme (143, 144). As a result, there is a significant decrease in sphingolytic activity, therefore these compounds are termed as FIASMA (36). The compounds differ markedly in molecular structure. A prediction of inhibitory capacity is available by a structure-property-activity relation (SPAR) model in order to specify the structural and physicochemical characteristics including variables referring to p*K*_a_, logP, as well as a factor depicting the steric hindrance of the most basic nitrogen atom of the compound modulating the free presentation of a protonated nitrogen atom at the inner lysosomal surface (145). It is noteworthy that these compounds are licensed for medical use in humans, are minimally toxic, and in use for a broad range of clinical indications, including the treatment of intensive care patients. There is a long list of FIASMA of ASM including amitriptyline, imipramine, desipramine, doxepine, fluoxetine, maprotiline, nortriptyline, paroxetine, sertraline, suloctidil, terfenadine, and famotidine, to mention a few (145) (Figure 1).

Furthermore, evaluating lysosomotropic and ASM-inhibiting activities in appropriate cell culture models are an applicable approach of newly designed substances to identify novel compounds with anti-apoptotic and anti-inflammatory capacity, which results ultimately in a decreased response of prototypic inflammatory mediators. At the end, in new candidate drugs, based on established SPAR models, physicochemical and biological properties will be selected and identified to enrich the pool of compounds, which might be beneficial for the treatment of the adverse effects of ASM upon activation (146).

In the next paragraph, there is a short overview on the proposed beneficial effects of ASM inhibition in a series of conditions associated with development of organ failure and long-term effects of sepsis sequelae, classified by the affected tissue.

### Muscles, Diaphragm—Heart, and Cardiovascular System

Cardiac dysfunction, in particular of the left ventricle, is a common and early event in sepsis and is strongly associated with an increase in patients' mortality. Surrogates of cardiomyopathy cardiac function, ceramide formation, markers of oxidative stress, as well as troponin I levels were found to be improved in FIASMA-treated animals in a semi-lethal peritonitis model (147). Interestingly, in this study, an activation of *de novo* synthesis of ceramides could be identified to be responsible for cardiac ceramide increase (147). In an *in vitro* and *ex vivo* experimental setting, Ferreira et al. showed that mouse myotubes and diaphragm muscle fiber bundles are sensitive to ASM treatment mediated by release of mitochondrial ROS, resulting in significant depression of diaphragm force and accelerated fatigue in a time and concentration manner (148, 149). Also, the p47(phox) subunit of NADPH oxidase is held responsible to play an important role on oxidant-mediated diaphragm weakness triggered by ASM (150). Similar results could be obtained in a chronic malfunction of skeletal muscles and dysregulation of sphingolipid turnover in insulin-responsive tissues at old age. In parallel with a progressive increase of ceramide content and Cer/SM ratio during aging of rats, there is a decrease of insulin responsiveness, which can be overcome by ASM inhibition (151). Interestingly, in muscle tissue, the counteracting activity of sphingosine-1-phosphate is once more evident, promoting cell survival, Ca^++^ mobilization, fiber growth and repair, as well as fatigue resistance [excellently reviewed in (152)].

ASMKO mice are protected from TNF-α-induced hypotension and tachycardia (153), which might be regarded as clinical symptoms of systemic inflammation (154). One hypothesis for this surprising observation might be a ceramide-triggered activation of NOS, resulting in vasodilation and hypovolemic shock (153), which was in a similar manner also observed for ceramide generation by the neutral isoform of sphingomyelinase (155). Therefore, pharmacological inhibition of ASM-triggered ceramide generation and presumed prevention of shock should be considered to contribute to the overall improved survival (153) in the acute phase as observed in a series of peritonitis models in mice (147, 156).

#### Lung

Pulmonary outcome following endotoxin challenge in mice was improved by ASM inhibition, resulting in attenuating alveolar collapse (157). Inhibition of ASM was identified as a possible target in acute lung injury and pulmonary edema, induced by administration of platelet-activating factor and endotoxin (158). Fluoxetine and desipramine reduced in ovalbumin-sensitized rats the number of migrated immune cells into bronchalveolar lavage fluid but did not exert anti-inflammatory activity by attenuation of bronchial hyperreactivity (134).

#### Liver Tissue—Long-Term Effects on Tissue Function and Fibrosis Following Sepsis

Long-term sepsis survivors might develop hepatocellular/hepatobiliary injury and fibrosis (159–162). ASM, also an important regulator of hepatocyte apoptosis and hepatic stellate cell (HSC) activation (163), is linked to the promotion of liver dysfunction in the acute phase as well as to fibrogenesis in the long term. In both, the acute and the postacute phase, pharmacological inhibition of ASM displayed a beneficial effect on oxidative stress levels, hepatobiliary function, macrophage infiltration, hepatic stellate activation, and overall survival (156). ASM inhibition exhibited a protective effect on liver function in the acute phase, and the reduction of HSC activation diminished development of sepsis-associated liver fibrosis in the postacute phase of sepsis (156). In this context, dysregulation of hepatic biotransformation capacity, especially of the cytochrome P450 (CYP) system, represents an important distress factor during host response (164–166). Thus, these enzymes are estimated to be responsible for metabolizing >75% of drugs which are in daily clinical use. Pharmacological inhibition of ASM has an important impact on expression and activity of different hepatic CYP enzymes using an animal model of polymicrobial sepsis in the acute as well as in the postsepsis phase (167).

In murine models of peritonitis and endotoxemia following LPS challenge, amitriptyline-treated mice were protected from overwhelming cytokine release (KC, MCP-1) and from pulmonary edema as well as exhibited improved survival. Anti-inflammatory effect of amitriptyline treatment is reflected by increased IL-10 levels and decreased accumulation of immune cells at the site of infection (135).

In all studies enrolling patients with severe infection, sepsis and multiple organ failure as well as in all reports modeling the disease continuum *ex vivo* or *in vivo*, we found a clear association of ASM activity with morbidity and unfavorable outcome. These observations are independent from the underlying disease such as peritonitis, pneumonia, and HHL. On the other hand, inhibition of ASM activity is capable to prevent destructive events of overwhelming immune activation in all affected tissues and circulating cells, be they nucleated or not. Here, we come to the point for further consideration of the presumed beneficial effects of ASM inhibition in daily clinical care, because a broad panel of drugs is exhibiting ASM inhibition.

## Conclusions and Further Perspectives

The expenditure for locally restricted or disseminated ceramide generation by ASM for effective host response is remarkable. The multifaceted roles of ceramide in this context, specific function in individual tissues and organs, the flux and interconnections of lipid mediators with agonistic functions requires further investigation and redefining. Nonetheless, there is conclusive preclinical evidence that the conserved stress enzyme ASM plays an essential role in the pathogenesis of sepsis/host response and that its inhibition might improve the outcome. However, the gap between preclinical and clinical trials has not been convincingly closed so far. Therefore, we suggest as a first step that a retrospective analysis of sepsis patients in the intensive care unit, who are coincidentally treated with FIASMAs, should be initiated to overcome the missing link from bench to bedside. Our knowledge gained from preclinical experiments indicates that FIASMAs might be promising candidates for future pharmacological studies targeting ASM in sepsis and host response keeping in mind that these drugs are already FDA approved and just need to be repurposed.

### FIASMA—From Unattended ASM Inhibition at ICU to an Intended Use

FIASMA are a class of drugs, which are widely used for (often chronic) treatment of a number of symptoms such as major depression, neuropathic pain, fibromyalgia, etc., to mention a few (168). From that list, major depression was recently ranked as the third leading burden of non-fatal diseases worldwide with a prevalence of >10% in all analyzed regions and with an increasing incidence to be first during the next decade (169, 170). In the *Global Burden of Disease* survey for 2017, more than 264 million cases were reported worldwide (169).

In Germany, the 12-month prevalence of major depression is 12% in adults, of whom the majority is treated with antidepressant drugs (171).

In cultured PBMC from healthy volunteers treated either with imipramine or amitriptyline in a therapeutical dosage, activity of ASM is rapidly reduced to levels around 30% of original activity over a period of 3 weeks. Interestingly, removal of the drugs resulted in a gradual and prolonged, but complete normalization of activity over a period of 5–6 days (172). Considering this scenario combined with the facts that (i) daily use medication is often or regularly spared on ICU admission and (ii) that drugs initially used at the newly admitted ICU patient might also exert an inhibitory capacity on ASM, it seems quite possible that the septic patients at least in the early days following admission might be subdivided in separate groups: one with decreased ASM activity due to antecedent treatment with FIASMA due to other indication and gradual normalization of ASM activity, one with inhibition of ASM activity only just at ICU submission, and one without any interference with ASM activity. In addition, it might be further speculated that the presented ASM activity in circulation is also superimposed by the conserved mechanisms of stress response as discussed in detail previously.

This scenario in mind, there are some principal opportunities, risks, as well as consequences examining host response and its unintended, but presumptive treatment by FIASMA.

1. For a more precise interpretation of results from clinical sepsis studies, the definition of the as-yet unknown biasing factor of ASM inhibition by consideration of both ASM activity and inhibition by FIASMA due to other indication prior to hospitalization is needed (anamnesis, documentation of prehospital medication, identification of FIASMA therein). In sepsis trials, depression at least (and treatment thereof) should be defined as a significant comorbidity.
2. The incidence of sepsis is determined including clinical parameters of organ dysfunction in a cohort undergoing prehospital treatment with FIASMA compared with those without. Answering this question will also result in the assessment, whether FIASMA-treated patients are at higher risk for a severe course due to putative impairment of phagolysosomal elimination of invading pathogens or—on the contrary—at lower risk for development of tissue damage and organ failure due to potential beneficial effects of FIASMA. From an epidemiological point of view, two studies came up with interesting results: in a general population (~60,000 individuals) with self-reported anxiety and depression symptoms, severe depression was found to be associated with an increased risk for blood stream infection, moderate were not. However, an increased mortality risk was found for the later subcohort only; unfortunately, no data on antidepressant treatment were given in this study (173). Similar results were obtained from a Danish cohort study, where the underlying depressive disorder was assessed either by psychiatric diagnosis or by at least two antidepressant prescription redemptions within a 6-month period prior to hospitalization due to sepsis (174).
3. On ICU, ASM activity is also determined in septic patients treated with FIASMA due to other indications, comparison with an untreated group, and association with clinical parameters.
4. Ultimately, there is also a need for placebo-controlled trial with a carefully selected FIASMA (on the basis of data from observational trials) for a prospective investigation of the proposed beneficial effect of resulting ASM inhibition with respect to severity (development, duration, resolution of organ failure) and overall outcome.

### Drug Repurposing

Repurposing of established drugs to treat both, common, or rare diseases is becoming an increasingly attractive and fast-track approach because it involves the use of compounds with known pharmacokinetic and safety profiles, with potentially lower overall development costs and shorter development timelines due to existing approval by the regulatory authorities (175). In the case of ASM, it is of remarkable importance since there is a long list of potential candidates available (36), and both, the structure-activity relationship (145) as well as the underlying mechanism of effective inhibition are known (144). Also, there are promising results from preclinical and clinical studies as discussed in this review, and there are some more outcomes using an explorative design for identification of beneficial effect of ASM inhibition.

Out of around 800 unique three-drug combinations, two sets were identified to effectively inhibit Ebola virus entry into human cells and were further validated for inhibition of live Ebola virus infection—at least two drugs of the triple exhibiting effective ASM inhibitory capacity (176).

There is also an excellent review [Beckmann et al., (177) and references cited therein], critically outlining the effectiveness of amitriptyline in a series of serious conditions such as cancer, infection, and metabolic and neurological diseases, all of them assessed as ASM-related diseases. For this drug, potential new applications for therapeutical treatment are demonstrated which might also be considered a general opportunity, since amitriptyline is proposed to function pars pro toto for this group of drugs exhibiting inhibitory capacity for ASM. Beside other drugs, these studies revealed that amitriptyline is a promising candidate for further consideration for the treatment of infectious diseases and overwhelming host response. However, adverse effects of a missing residual activity as shown by incompetence for phagocytosis and increased bacterial burden were also observed (65, 81). On the other hand, there is a clear association of ASM activity with the severity of sepsis and unfavorable outcome (34, 113, 128). It has to be mentioned that in heterogeneous mice by FIASMA treatment, a temporary status similar to complete loss of function was shown, underlining the effectiveness of functional ASM inhibition (167). A residual activity of 15% is insufficient to prevent clinical features of Niemann-Pick diseases over time (17).

It is of major interest that fluoxetine, a widely used antidepressant drug, efficiently inhibited the entry and propagation of SARS-CoV-2 in the cell culture model without cytotoxic effects and also exerted potent antiviral activity against two currently circulating influenza A virus subtypes, an effect which was also observed upon treatment with the FIASMAs amiodarone and imipramine (178).

The recently proposed concept of a stress/injury-induced lysosomal exocytosis (59) as the major source of extracellularly circulating ASM (38) is in line (i) with a restoration of dependency for Zn^++^ ions, since due to rapid displacement of these ions after reaching the extracellular space, an additional regulating factor participates in the resolution of ASM activity beyond the outfall of the lysosomal content, and (ii) the effectiveness of activity decreases in circulation upon treatment with FIASMA, resulting in intralysosomal proteolysis of the mature ASM protein and subsequent release of inactive protein fragments without sphingolytic activity, and (iii) missing evidence of phosphorylated ASM in circulation of a patient undergoing stress response. However, the validation of this concept in septic patients still needs further examination, e.g., by comparison of the fragmentation and phosphorylation pattern of ASM isolated from both compartments, lysosomes and plasma, obtained from patients undergoing treatment with FIASMA. Another important point with respect to the dosage of FIASMA for treatment of host response is the fact of similarity to that of the original indication (often major depression) due to a very similar mode of action regarding ceramide generation.

### Résumé and Take Home Messages

During an episode of sepsis, a broad panel of cell, tissue, and organ response is controlled by stress-induced ceramide generation; there is a broad understanding of potentially harmful effects of ceramide generation during sepsis and there is a broad panel of well-established and approved drugs with effectiveness for ASM inhibition (Box 2), at the end encouraging systematic studies for detailed examination of an unattended or an intended inhibition of ASM during sepsis to improve patients outcome.

Box 2 **Take Home messages.**

- ASM activity in circulation is a marker and mediator of harmful events, increased in patients with sepsis and associated with the clinical course regarding development, duration, and resolution of organ failure and outcome;
- In septic patients, different phenotypes with respect to ASM activity might be expected due to antecedent treatment with drugs for other indications (i.e., major depression etc.), which are also inhibiting ASM by lysosomotropic mechanisms;
- Further investigation in upcoming clinical studies is necessary to examine the potential consequences of an unattended ASM inhibition;
- Intended inhibition of ASM, also by repurposed drugs, is a promising approach to control the adverse effects of an overwhelming ASM activity associated with unfavorable outcome in these patients.

### Methods

The objective of this narrative review is to evaluate the changes of sphingomyelinase activity during infection and host response and the discussion of translation into the field of translational sepsis research including development of new strategies for diagnosis and treatment. A search in the main biomedical databases (PubMed, Medline, Scopus, and Web of science) was conducted for a 20-year period ending in July 2020, focused on primary research articles in the field of interest. Keyword search for abstracts and titles included (“sphingomyelinase” OR “SMPD1”) AND (“sepsis” OR “inflammation” OR “infection”). The search identified 723 references, which were selected by particular importance for this review. The prescreened references were completed by other publications related to the issue. We apologize if a valuable work of any appreciated colleagues could not be included due to space limitations and the narrow scope of this review.

## Author Contributions

RC and H-YC drafted the manuscript. Both authors revised the final version of the manuscript.

## Conflict of Interest

The authors declare that the research was conducted in the absence of any commercial or financial relationships that could be construed as a potential conflict of interest.
